# Effect of Tronclass combined with team-based learning on nursing students’ self-directed learning and academic performance: a pretest-posttest study

**DOI:** 10.1186/s12909-024-05741-3

**Published:** 2024-07-12

**Authors:** Longyi Hu, Siqi Li, Leshan Zhou

**Affiliations:** Central South University Xiangya School of Nursing, Changsha, Hunan China

**Keywords:** Team-based learning, Self-directed learning, Nursing students, Visual teaching

## Abstract

**Background:**

Self-directed learning ability is crucial for lifelong learning. The inadequate self-directed learning ability of nursing students in China may be due to their deficiency in self-management or learning initiative.

**Objective:**

To explore the effect of online learning platform (Tronclass) combined with team-based learning on nursing students’ self-directed learning and academic performance.

**Design:**

Pretest-posttest design.

**Participants:**

From March to July 2023, 69 undergraduate third-year nursing students from a university in Hunan Province were selected through a whole-group sampling method.

**Methods:**

This study used Tronclass to carry out team-based learning in the teaching process of pediatric nursing courses. It compared the self-directed learning ability nursing students before and after courses, and juxtaposed their academic performance with those of their counterparts who graduated in previous years.

**Results:**

When comparing compare motivation, self-management, teamwork and information literacy, which are four subscale aspects of the self-directed Learning Ability Scale, the post-survey scores for these four dimensions are greater than the pre-survey results. The results of the study showed a statistically significant difference (*P* < 0.05), in the students who engaged in Tronclass combined with team-based learning. Specifically, these students received higher midterm and final grades than to those who had already graduated and did not participate in these activities. (*P* < 0.05).

**Conclusion:**

Combining Tronclass with team-based learning enhances nursing students’ ability to engage in self-directed learning and improves their performance in midterms and finals, thereby fostering the development of comprehensive competence.

## Background

Due to the healthcare system reform and the advancement of nursing education in China, nurses are now required t to possess more than only technical proficiency. Nursing educators have also focused on cultivating students’ comprehensive abilities, such as empathy, self-directed learning, and scientific research [1–4]. Self-directed learning (SDL) refers to an individual’s initiative in understanding what they should work on, establishing their learning goals, selecting and implementing appropriate learning strategies, and evaluating learning outcomes, with or without help from others. Nurses need to learn new skills and knowledge, and SDL reflects the nursing students’ initiative and is important to lifelong learning [5]. Zhou et al. [6] surveyed 2,438 undergraduate students in China on their SDL and found that 30.7% had weak SDL ability and might lack confidence in their capabilities or self-management ability, Meanwhile, 35.7%were unwilling to seek help or obtain nursing information.

Traditional nursing education is all about “chalk and talk” teaching. Teachers are the absolute leaders while students are mere participants who are difficult to mobilize [7]. Learning through online platforms has become a new trend, as the Internet emerges, Chen et al. [8] analyzed nine articles on network learning and found that students were more motivated to learn independently using online platforms. Students chose time, set goals, and made plans more aligned with their developmental needs. It upgraded students’ self-management skills, but reduced communication between students to the detriment of teamwork.

Team-based learning, proposed by the management scientist Parmelee [9], is referred to as “an active learning and small group instructional strategy that provides students with opportunities to apply conceptual knowledge through a sequence of activities that includes individual work, teamwork, and immediate feedback.” It not only plays to students’ subjectivity but also exercises students’ communication skills, prompts students to learn from each other, and improves their efficiency [10]. However, it suffers from an uncontrollable division of labor among team members, and there may be situations where someone does not participate at all or where only one team member completes the whole project [11].

Considering the disadvantages of the two teaching methods, they can be complementary. This study intends to carry out team-based learning in the teaching process of pediatric nursing courses through an online learning platform (Tronclass) to overcome the drawbacks of online platform learning, where students usually learn behind closed doors and cooperative learning where uncontrolled division remains a problem, and to improve students’ SDL ability and academic performance.

## Design

This study was based on a one-group pretest-posttest design. It compared the differences in nursing students’ SDL ability before and after the course and the differences in mid-term and final academic performance with senior students who had already graduated.

### Participants

Sample size: The test was conducted using G*power, setting η^2^ = 0.06, α = 0.05 and1-β = 0.95, calculating a total sample size of at least 54.

A total of 69 undergraduate third-year nursing students from a university in Hunan Province were selected from March to July 2023 through a whole-group sampling method, meeting the predetermined minimum sample requirement of 54 participants.

**Fig. 1:**
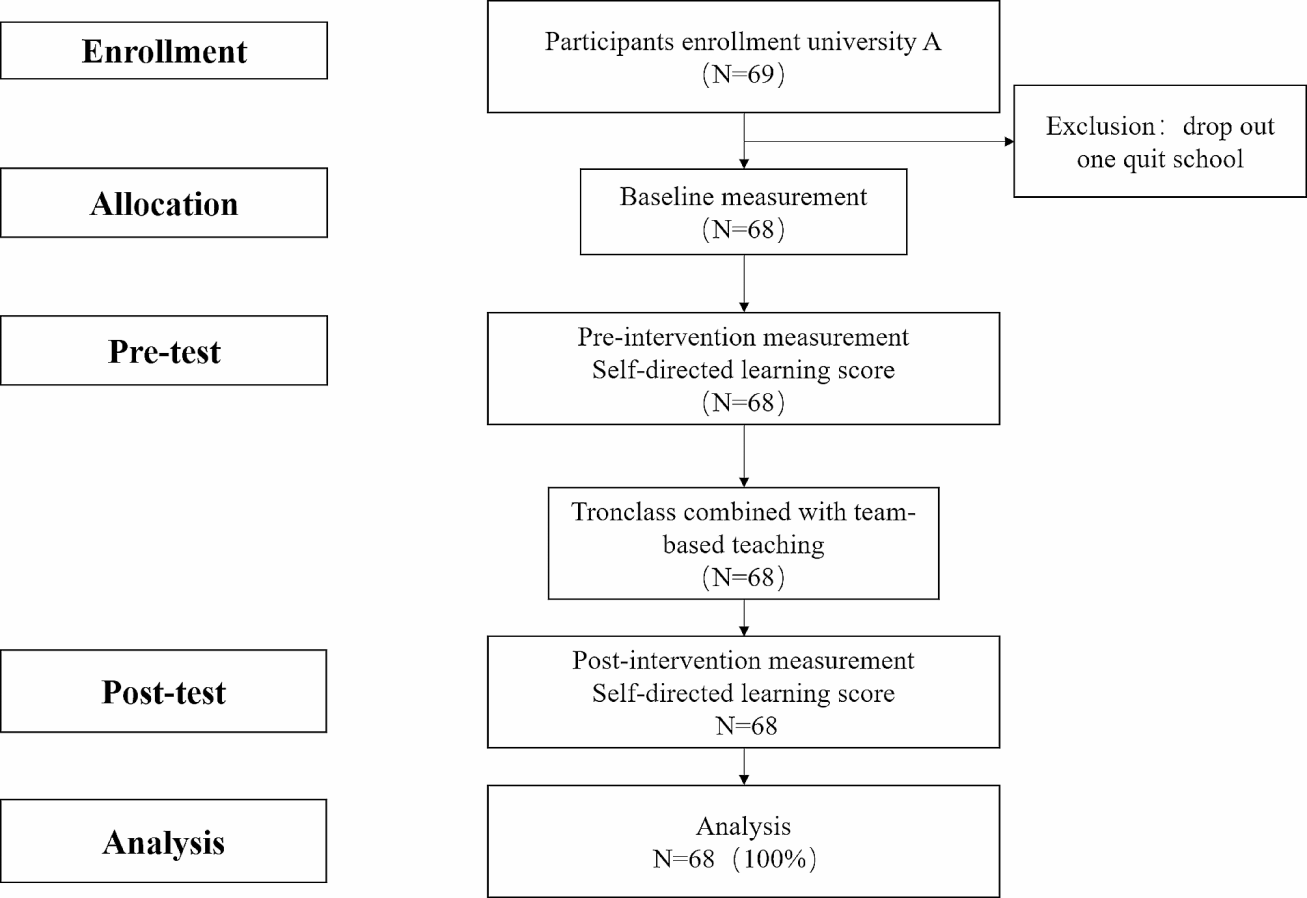
Flow diagram of the participants of this study Effect of Tronclass combined with team-based learning on nursing students’ self-directed learning and academic performance

Inclusion criteria: (1) Full-time nursing undergraduates; (2) Ability to read and comprehend; (3) Informed and consent to participate in this study; Exclusion criteria: (1) Students with psychosomatic abnormalities; (2) Students who was suspended, withdrew from school or joined halfway. One out of 69 Participants dropped out of the program, and data from the rest 68 were collected. Figure 1 shows flow diagram of the participants of this study.

### Ethical considerations

Before data collection and recruitment, the Ethics Review Committee approved this study. Participants were given a detailed explanation of the study and informed that they would not receive any credit for participating.

### Curriculum design

The textbook was the seventh edition of《Pediatric Nursing》. We added Tronclass (TC) combined with team-based learning to traditional teaching. TC, a “visual teaching platform,” developed by our university, is best used with operating systems Win 7 or higher and MAC OS X. Compatible browsers include IE 10, Edge, Chrome 55, Firefox 50.0, and Safari 9.0 or above. The platform also has a mobile application. Various functions are available on either PCs or mobile phones. This study, primarily used the platform to upload teaching resources, time-limited tests, anonymous grading, etc.

#### TC conducts chapter tests

Teachers uploaded the videos of each chapter to TC before class. Teachers post reflection questions via TC text, pictures, audio-visual, etc. Students independently learned simple chapters, such as Disease Overview, Child Growth and Development, Newborn Care, and other chapters that focusing on comprehension and memorization, and helped each other in teams. Teachers taught difficult chapters offline, such as systemic diseases, and fluid therapy. A time-limited test was issued after students finished one chapter to assess their understanding and application of what they had learned. Each test consisted of 10 multiple-choice and judgmental questions designed to test memorization, comprehension, and analysis ability. Each student had a different order of exam questions or options, with a time limit of 5 min. Only scores, but no correct answers, were released after the tests. TC would give students a score after they finished a test. It would also calculate the error rate and distribution of options for each question. The answers and explanations were published through TC after completing a whole chapter.

#### TC conducts team-based learning

The classes were divided into four groups and the assignments were accomplished through teamwork. Students used TC to score assignments anonymously. Teacher scoring comprised 60% of the final grade, anonymous scoring by teammates in the same team 20%, anonymous scoring by teammates to other groups after discussion 20%.

1) Health education: Based on our knowledge of pediatric nursing and public needs, we drafted and published a We chat push, in the hope that students could divide the work and urge group members to complete it together. Assignments were given at the beginning of the course. Students completed the work after class, and submitted WeChat Push by the end of the course.

2) Role-playing: Each group created a scenario, based on pediatric nursing, and corresponded to the clinical patient scenarios. The theme was chosen by the students. Students played different roles and acted out the performances of the different characters.

### Data collection

We gathered basic participant information and assessed SDL ability before the course and assessed SDL ability again after the course. Comparing nursing students’ SDL ability before and after the course as main outcomes and mid-term and final academic performance with previous students as secondary outcomes.

1) General information included: gender, age, only child or not, residence, father’s/mother’s occupation, participation in student clubs, study styles, learning styles, the reason for choosing the nursing profession, liking the nursing profession and willingness to be employed.

2) SDL ability: The scale was compiled by Zhang et al. (Zhang et al., 2009), and consisted of four subscales: motivation, self-management, teamwork, and information literacy, with 30 entries, scored 30 ∼ 150 using the 5-point Likert scale. Higher scores meant higher SDL ability with Cronbach’s α being 0.82.

3) Mid-term and final academic performance: The same teaching team created the tests. The contents, question types, and difficulty of the assessment were the same. The results are based on a percentage system.

### Statistical analysis

All analyses were performed using SPSS 25.0. Scores were calculated for descriptive demographics and characteristics, are presented as numbers, percentages, means, and standard deviations (SD) for normally distributed variables and as interquartile ranges (IQRs) and medians for non-normally distributed variables.

A paired t-test was used to compare SDL ability scores, and a two- independent samples t-test was used to compare mid-term and final academic performance.

## Results

### Characteristics

A total of sixty-eight questionnaires were distributed with a 100% return rate. Of all participants, 52(76.5%) were female and 16 (23.52%) were male a Table 1 depicts the general information used in this study.

**Table 1:** Characteristics (*N* = 68)

	*N*(%) / Mean (SD)
Gender	
Male	16(23.52)
Female	52(76.47)
Age	21.10(0.694)
Only child	
Yes	27(39.71)
No	41(60.29)
Father’s occupation	
Medical-related	1(1.47)
Non-medical related	67(98.53)
Mother’s occupation	
Medical-related	3(4.41)
Non-medical related	64(94.12)
Family residence	
City	34(50.00)
Townships	15(22.06)
Village	19(27.94)
Participation in student clubs	
Yes	38(55.88)
No	30(44.12)
Study styles	
independent	38(55.88)
Co-operative	4(5.88)
both	26(38.24)
Learning mode	
visual	45(66.18)
auditory	13(19.12)
tactile	10(14.71)
Reasons for study nursing	
independent choice	23(33.82)
Suggestions from others	26(38.24)
Speciality Transfer	19(27.94)
Be a nurse after graduation	
Very like	6(8.82)
A little like	42(61.76)
Not sure	15(22.06)
A little dislike	3(4.41)
Very dislike	2(2.94)

### SDL ability

Results showed that post-survey SDL ability and scores on all dimensions were better than pre-survey(*P*<0.05). See Table 2.

**Table 2:** Comparison of SDL ability pre- and post-survey

Items	Pre-(Mean ± SD)	Post-(Mean ± SD)	Δ	T	*P*
SDL	104.84 ± 10.75	114.19 ± 10.15	9.35 ± 12.13	6.358	<0.001
Motivation	28.38 ± 4.52	29.96 ± 3.37	1.57 ± 5.04	2.577	0.012
Self-management	38.66 ± 4.71	42.40 ± 4.01	3.74 ± 4.94	6.239	<0.001
Teamwork	16.99 ± 1.93	19.53 ± 1.94	2.54 ± 2.65	7.931	<0.001
Information literacy	20.81 ± 2.49	22.31 ± 2.57	1.50 ± 3.43	3.604	0.001

### Mid-term and final academic performance

Students who participated in TC and team-based learning achieved greater scores on their midterm and final exams than students from previous years (*P*<0.05). Table 3 presents a detailed analysis.

**Table 3:** Comparisons of mid-term and final academic performance

	Group	*N*	Score(Mean ± SD)	T	*P*
Mid-term	previous	59	70.81 ± 13.23	5.939	<0.001
	Current	68	84.12 ± 12.02		
final	previous	59	68.93 ± 13.02	8.230	<0.001
	Current	68	77.16 ± 12.36		

## Discussion

### TC combined with team-based learning improves nursing students’ SDL ability

Results showed that post -survey SDL ability and scores on all dimensions were better than pre-survey.

Transforming motivation fundamentally strengthens students’ SDL ability. Role-playing is widely used in nursing teaching and can stimulate nursing students’ interest in learning using scenario-based simulation [12]. This course reinforces student autonomy, and it was written and directed by the students in teamwork from real life or clinical cases rather than by the teacher. Wang et al. [13] argued that role-playing transforms passive learning into active learning in a way that changes students’ motivation. Health education was another means to change motivation. Nursing students often absorb the information presented by their lecturers, but in health education, role reversal allows, students to assume the role of educators rather than simply receiving knowledge passively. The change in learning motivation has moved from passive reception to active education, leading to an increased sense of ownership and initiative.

Self-management is the foundation of SDL. The advent of the Internet has increased the level of flexibility for nursing students. Chen et al. [14] conducted a study involving 245 undergraduate nursing students. The researchers discovered that those who studied online could review the videos of chapters they did not understand based on their situation, and these students exhibited better self-management skills. Rønning et al. [15] conducted a literature analysis and found that role-playing made nursing students understand better, while subsequent reflection allowed them to identify and address any areas of deficiency. Cooperative skills are another manifestation of SDL. Role-playing requires the application of knowledge. Students usually do their pre-study for better play. Furthermore, Barton et al. [16] reviewed 19 articles on teamwork, and found that students were virtually debriefed during role-playing through co-observation, guided questioning, and scenario analysis. Nursing students discuss and exercise their communication and teamwork skills. While completing the education program, nursing students need to assign tasks independently, and each of them will use their different knowledge to solve the problem, and maximize the advantage of teamwork.

Improved information literacy reflects enhanced independent learning skills. Nursing students incorporate what they do, feel, and think in the scenario simulation. They observed their lives, extracted information them, used online resources well, and looked for literature to create scenarios. Students gather information from the classroom, the practical process and the Internet to independently determine directions of interest during health education. Nursing students reviewed the literature independently summarized relevant evidence, and found the best evidence for scientific health education. As children are a special group of people, the audience of the health education program includes children and their parents. Nursing students need to articulate their words clearly in an easy-to-understand way, depending on the characteristics of the audience.

TC played a crucial role throughout the course. TC had a website and mobile application. Students can utilize fragmented time to study anytime, anywhere, which facilitates learning for nursing students [17]. TC tasks, such as pictures and audiovisuals, are in various forms to keep nursing students interested. In team-based learning, while mutual ratings can reduce the likelihood of large gaps in the division of labor among the team, the non-anonymous format may not be objective. TC has an anonymously rating system, and mutual scoring among group members can encourage nursing students to take the initiative to learn and reduce the possibility of an unbalanced division of labor.

### TC combined with team-based learning improves nursing students’ academic performance

Students who used the TC platform and practiced team-based learning achieved higher midterm and final grades compared to their seniors who hadn’t done so. TC sets each student’s exam questions and choices differently during classroom tests, which preventing students from copying others’ answers and urging nursing students to learn independently Team-based learning improves nursing student learning accomplishments in two aspects. On the one hand, nursing students are more attentive to learning what they are responsible for in their team tasks [18]. Sudents communicate with each other in team-based learning, share relevant information, and improve information literacy, promoting knowledge comprehension. Allert et al. [19] interviewed 11 students and found that when students explain what they learned in class to each other, both the explainer and the questioner understand better. Furthermore, nursing students acquire knowledge about the advancement of other groups through TC and gain insights into how other groups study to enhance their SDL ability.

### Application and implications of TC combined with team-based learning

Online platform learning helps students utilize online learning resources better and nursing students are more active. However, nursing students need strong self-management abilities. Independent learning may weaken cooperation and communication abilities. Conversely, team-based learning, improves cooperation, but team division of labor is uncontrollable. This study combines the two approaches to pediatric nursing.

We used the TC anonymous scoring system to avoid an unbalanced division of labor. Team-based learning promotes information exchange between students. The combined way of teaching improves nursing students’ SDL and academic performance. The auto-scoring and analyzing aspects of TC allowed teachers to give specific solutions depending on scoring details. Furthermore, it minimized unnecessary tasks for teachers and allowed them to allocate more time to instruction. TC is user-friendly and does not necessitate advanced hardware infrastructure. Other educational teams can also apply similar platforms to accomplish instructional objectives, and it is deserving of endorsement.

### Limitations of this study

The present study has certain limitations. First, parallel control groups were not set up in this study because it is inappropriate to group the students considering their number and grouping might lead to contamination effects and pedagogical and ethical issues. Second, faculty competence, teaching conditions, and individual students varied from school to school. Participants in this study were from the same university and did not represent all nursing students.

## Conclusion

This study combined team-based learning and the TC method in the pediatric nursing course. As a result, the nursing students demonstrated increased autonomy and motivation through SDL and teamwork. This improvement in SDL and academic performance was observed among the nursing students, enhancing the efficacy of online platform learning.

## Data Availability

The datasets analyzed during the current study are not publicly available, but are available from the corresponding author on reasonable request.
